# Photometric Compliance of Tablet Screens and Retro-Illuminated Acuity Charts As Visual Acuity Measurement Devices

**DOI:** 10.1371/journal.pone.0150676

**Published:** 2016-03-22

**Authors:** I. A. T. Livingstone, C. M. Tarbert, M. E. Giardini, A. Bastawrous, D. Middleton, R. Hamilton

**Affiliations:** 1 Department of Ophthalmology & Glasgow Centre for Ophthalmic Research, NHS Greater Glasgow & Clyde, Glasgow, United Kingdom; 2 College of Medical, Veterinary and Life Sciences, University of Glasgow, Glasgow, United Kingdom; 3 Department of Clinical Physics, NHS Greater Glasgow & Clyde, Glasgow, United Kingdom; 4 Department of Biomedical Engineering, University of Strathclyde, Glasgow, United Kingdom; 5 International Centre for Eye Health, Clinical Research Department, London School of Hygiene and Tropical Medicine (LSHTM), London, United Kingdom; University of Copenhagen, DENMARK

## Abstract

Mobile technology is increasingly used to measure visual acuity. Standards for chart-based acuity tests specify photometric requirements for luminance, optotype contrast and luminance uniformity. Manufacturers provide some photometric data but little is known about tablet performance for visual acuity testing. This study photometrically characterised seven tablet computers (iPad, Apple inc.) and three ETDRS (Early Treatment Diabetic Retinopathy Study) visual acuity charts with room lights on and off, and compared findings with visual acuity measurement standards. Tablet screen luminance and contrast were measured using nine points across a black and white checkerboard test screen at five arbitrary brightness levels. ETDRS optotypes and adjacent white background luminance and contrast were measured. All seven tablets (room lights off) exceeded the most stringent requirement for mean luminance (≥ 120 cd/m^2^) providing the nominal brightness setting was above 50%. All exceeded contrast requirement (Weber ≥ 90%) regardless of brightness setting, and five were marginally below the required luminance uniformity threshold (L_min_/L_max_ ≥ 80%). Re-assessing three tablets with room lights on made little difference to mean luminance or contrast, and improved luminance uniformity to exceed the threshold. The three EDTRS charts (room lights off) had adequate mean luminance (≥ 120 cd/m^2^) and Weber contrast (≥ 90%), but all three charts failed to meet the luminance uniformity standard (L_min_/L_max_ ≥ 80%). Two charts were operating beyond manufacturer’s recommended lamp replacement schedule. With room lights on, chart mean luminance and Weber contrast increased, but two charts still had inadequate luminance uniformity. Tablet computers showed less inter-device variability, higher contrast, and better luminance uniformity than charts in both lights-on and lights-off environments, providing brightness setting was >50%. Overall, iPad tablets matched or marginally out-performed ETDRS charts in terms of photometric compliance with high contrast acuity standards.

## Introduction

The widespread use of mobile technology is generating innovative ways to improve health. Globally, it is estimated that 500 million mobile device users will download healthcare applications (‘apps’) by 2015 [1]. This brings an opportunity to enhance detection of compromised vision. With today’s knowledge, technology and treatment, an estimated 80% of global blindness is preventable or curable [2] and mobile technology-based approaches represent a credible means of radically changing how we detect visual disability [3,4]. It is therefore unsurprising that mobile technology, in particular the iPad tablet (Apple Inc., CA, USA), has been evaluated as an alternative to traditional chart-based methods for measuring vision [5–14]. However, only a minority of apps presently available have been subject to robust evaluation, and the number of apps is rising: in 2012, 32 iPhone (Apple Inc., CA, USA) apps purported to assess visual function [15], while currently (22/07/2015), the search phrase “vision test” in Apple’s online app store (www.store.apple.com/uk) generates 151 results. As with more traditional medical devices, apps used in a clinical context, and the platform or device on which they run [16], require regulation because of potential risks to the public. Updated guidance regarding which applications and target platforms are appropriate for clinical use is needed, and regulation, accreditation and ‘kitemarking’ of various healthcare technologies including apps is planned in the UK [17].

High contrast visual acuity assessment remains the key, measurable outcome for defining abnormal vision and mapping changes in visual function, and has been widely targeted for apps on mobile devices [3–5,7,8]. Both optotype contrast and test luminance affect acuity measurements [18,19], and therefore devices purporting to measure acuity for clinical purposes should be standardised as specified for chart tests. Chart luminance should be ≥ 80 cd/m^2^ [20] or ≥ 120 cd/m^2^ [21], depending on the standard used. The ETDRS specification of 160 cd/m^2^ is based on the International Council of Ophthalmology (ICO) recommendations [20]. An evidence-based recommendation of 80–320 cd/m^2^ [18] was adopted for a non-clinical international standard [22]. Contrast specification also differs by standard: the ICO standard specifies that black optotypes should be ≤ 15% of the luminance of the white surrounding field [20], while BS 4274–1:2003 specifies that luminance (Weber) contrast [(L_bkg_−L_letter_) / L_bkg_] should be ≥ 90% [21]. Luminance uniformity is specified only in BS 4274–1:2003, which requires that any variation across the chart should be ≤ 20% [21].

Previous work has demonstrated suitable physical screen characteristics of three tablets from different manufacturers for contrast vision testing, providing individual device gamma functions are known [6,12]. The authors also noted that luminance uniformity varied within and between devices, but with little detriment to contrast uniformity [12]. This current study aimed to develop this work for high contrast acuity testing. It is likely that gamma functions matter less for high contrast acuity, and given the need for simple but sensitive point-of-care test procedures, more complex calibration procedures are not desirable. We investigated seven tablets (iPads, Apple Inc., CA, USA), measuring luminance, contrast and luminance uniformity and the effect of adjusting brightness settings. The tablets selected allowed comparison within the same device generation as well as across three generations of the currently available iPad range. We also assessed luminance, contrast and luminance uniformity of three gold standard ETDRS visual acuity charts, and compared these with the tablet findings. The effect of room lighting (on or off) was investigated for a subset of tablets and all three charts.

## Materials and Methods

**Fig 1:**
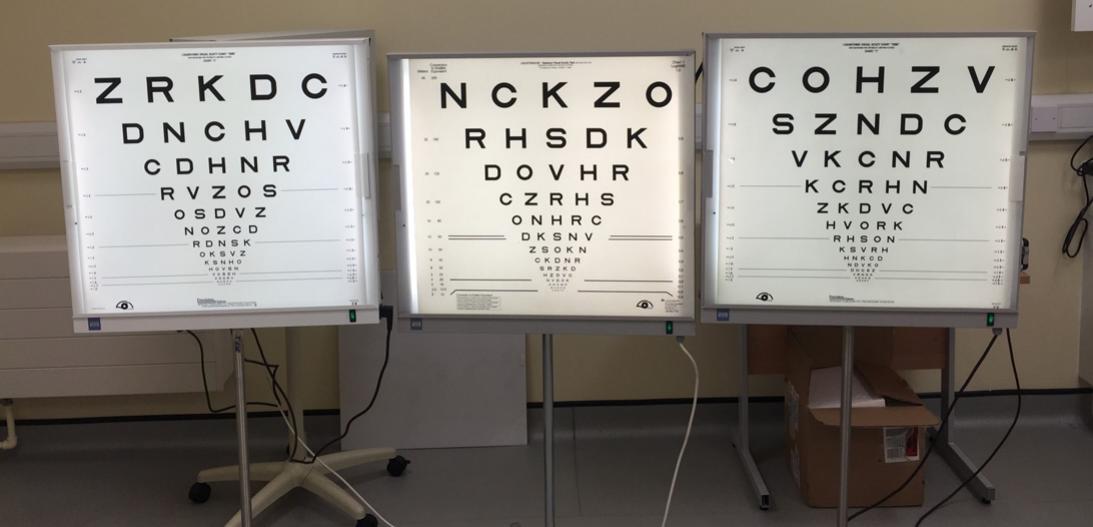
The three ETDRS charts.

Seven tablets were evaluated (one iPad 3, three iPad 4s, three iPad Air 2s, Apple inc, California, USA) along with three ETDRS charts (Precision Vision, IL, USA) mounted in separate illumination cabinets (Sussex Vision, Rustington, UK) (Fig 1). Charts and illumination cabinets varied in age, but were all in clinical use. Luminance measurements were performed using a Minolta LS-100 luminance meter (Konica Minolta Sensing, Europe B.V.) with a 1° aperture and a No. 110 close-up lens with a current calibration traceable to the German national standard. The procedure was similar to those recommended by national and international protocols [22,23].

Each tablet was placed on a horizontal surface with the luminance meter held in a tripod and positioned perpendicular to the screen. The meter was brought into focus on the position of interest and the distance between the screen and lens measured at 55 +/- 5 mm. For optimum screen stability, the tablet devices were switched on at least 15 minutes prior to data acquisition [6,12]. Auto-brightness was deactivated. Ambient light levels were measured with the Iso-tech ILM-01 light meter (RS Components, UK). Room lights were switched off to avoid reflections and stray light [22], with ambient light levels measuring 0.52 lx. To emulate real-life test conditions, three of the tablets were also measured with the room lights on, where ambient light levels measured 762 lx.

**Fig 2:**
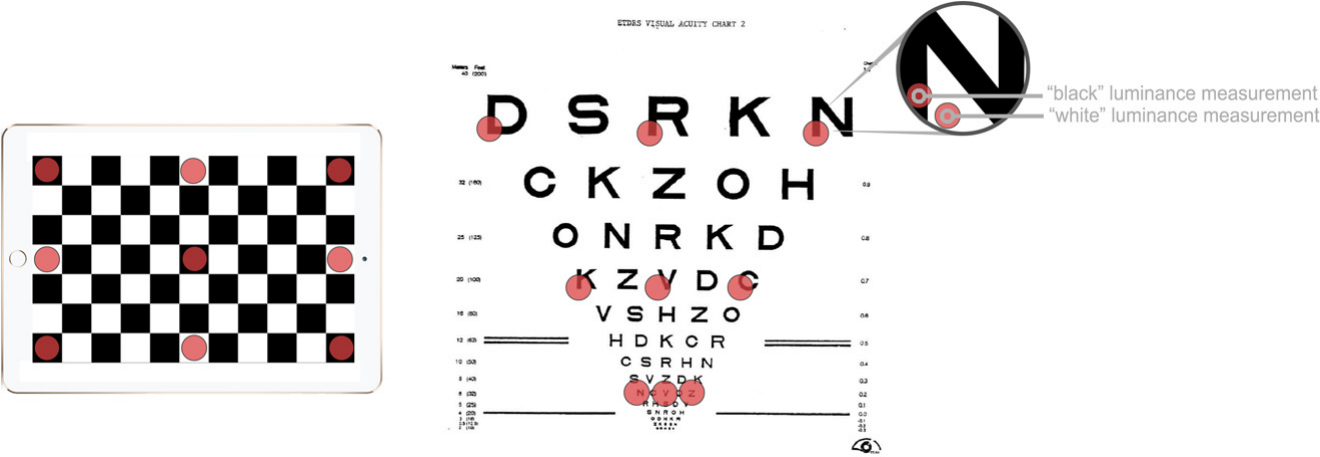
Illustration of cardinal points (red dots) where measurements were made for the tablets (left) and for the EDTRS charts (right).

A two-frame reversing black and white checkerboard test screen was created in Adobe Photoshop CC v14 (Adobe Systems Inc., CA, USA) with 186×186 pixel black (RGB 0 0 0) and white (RGB 255 255 255) squares. This allowed both maximum and minimum luminance measurements at the same screen location. Measurements were made at nine cardinal points (Fig 2), moving the screen with respect to the photometer between each point to maintain the perpendicular aspect [23]. Five levels of screen brightness were used based on linear position of the brightness setting slider bar, measured with a ruler. Minimum (“0%”) and maximum (“100%”) used the extreme positions, and three evenly spaced intermediate positions were used, hereafter labelled “25%”, “50%”, and “75%”. A manual brightness setting method was chosen over software-based methods, so if found necessary, brightness settings could be easily changed by health care professionals without dependence on bespoke software.

ETDRS charts were switched on for at least one hour prior to taking measurements. The luminance meter was hand-held 60 +/- 5mm perpendicular to the chart and focused on the lower left aspect of the black target letter, and on its adjacent white background. Room lights were switched on throughout to emulate routine clinical use: there is no guidance regarding calibration with or without additional room lighting. We therefore repeated the measurements on all charts with the room lights off.

For the seven tablets, each at five different brightness settings, and for the three EDTRS charts, overall luminance was calculated as the mean of the nine measurements across the screen or chart. Contrast was calculated for each of the nine cardinal points of the tablets by comparing luminance when black (L_black_) with luminance when white (L_white_), and for each optotype of the charts by comparing luminance of letters (also L_black_) with adjacent white background (also L_white_). Michelson contrast [(L_white_-L_black_) / (L_white_+L_black_)], a simple contrast ratio (L_black_/L_white_), and Weber contrast [(L_white_-L_black_) / L_white_] were calculated. The simple contrast ratio was calculated to determine compliance with the ICO standard which requires a ratio of ≤ 15% [20], and Weber contrast was calculated to determine compliance with BS 4274–1:2003, which requires a Weber contrast ≥ 90% [21]. Luminance uniformity was calculated as the ratio of the lowest of the nine white measurements to the highest (L_min_/L_max_) and used to determine compliance with BS 4274, requiring a ratio ≥ 80% [21].

Pilot testing established that variation of luminance within the black/white squares and within the chart letters/proximal background were minimal. Similarly, varying the focal distance of the luminance meter within ± 5 mm had negligible impact on luminance values.

## Results

### Luminance and contrast

**Fig 3:**
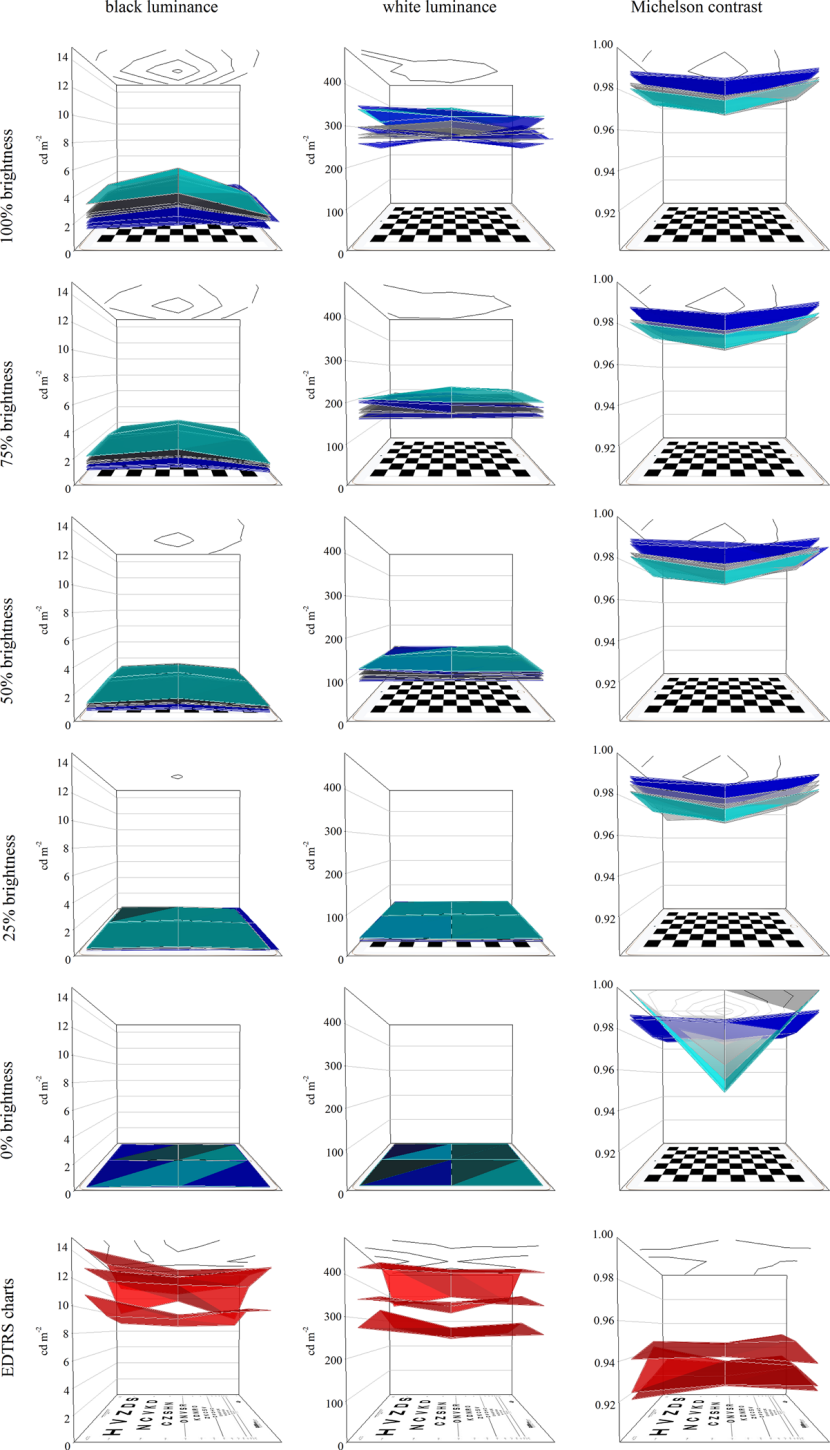
Luminance of black (left) and white (centre) squares, and of Michelson contrast (right) for the seven tablets at nominal maximum (“100%”, top) to minimum (“0%”, bottom) brightness settings. Coloured surfaces in each plot also demonstrate luminance and contrast uniformity, by joining the nine measured values. Ceiling contour plots represent the mean uniformity profile of all seven tablets. Cyan surface: iPad 3 tablet. Grey surfaces: iPad 4 tablets. Blue surfaces: iPad Air 2 tablets. Data for the three EDTRS charts based on luminance of black optotypes and adjacent white backgrounds are shown in the lowermost plots (red surfaces).

For all seven tablets, mean luminance of the display decreased as the brightness setting of the tablet was reduced, as expected. For white squares, luminance dropped from around 300 cd/m^2^ at the maximum setting to around 3.5 cd/m^2^ at the minimum setting, while black square luminance dropped from around 2–3 to 0.02 cd/m^2^ (Table 1, Fig 3). Mean Michelson contrast remained almost unchanged with brightness setting (97.9–99.6%). At minimum (“0%”) brightness, the luminance of the black squares measured zero in one case, created a spurious 100% contrast (Table 2, Fig 3, bottom right panel). Newer generations of tablet had lower black square luminance (“blacker blacks”) than older generations: iPad Air 2 black squares were typically 0.2 log units less bright than iPad 4 black squares. White square luminance changed much less: typically iPad Air 2 white squares were 0.02 log units less bright than iPad 4 white squares. Consequently, contrast was slightly higher for the iPad Air 2 tablets: ignoring values for the minimum (“0%”) brightness, iPad Air 2 Michelson contrast was 98.7–98.8% for all devices and four brightness settings, while iPad 4 Michelson contrast was 97.9–99.2% (Table 2).

**Table 1:** Luminance values (cd/m^2^) for seven tablets at five different nominal brightness settings. Values are the mean of the nine values measured across the screen for black and for white squares. Measurements were made with room lights off. Device/setting combinations for white squares which do not meet the BS 4274 requirement (≥ 120 cd/m^2^) are in bold, and those which do not meet the ICO requirement (≥ 80 cd/m^2^) are underlined.

nominal brightness setting	100%		75%		50%		25%		0%	
	black	white	black	white	black	white	black	white	black	white
iPad 3	3.3	331	2.0	202	1.2	**118**	0.37	**36**	0.03	**3.9**
iPad 4 #1	2.8	287	1.7	178	1.1	**111**	0.34	**33**	0.02	**3.6**
iPad 4 #2	2.6	278	1.6	165	0.9	**100**	0.28	**28**	0.01	**3.4**
iPad 4 #3	2.8	300	1.7	182	1.0	**106**	0.30	**33**	0.00	**3.6**
iPad Air 2 #1	2.0	315	1.2	181	0.7	**108**	0.21	**32**	0.02	**3.5**
iPad Air 2 #2	1.8	270	1.2	179	0.7	**110**	0.21	**32**	0.02	**3.3**
iPad Air 2 #3	1.9	281	1.1	166	0.6	**96**	0.20	**30**	0.02	**2.8**

**Table 2:** Contrast values (%) for seven tablets at five different nominal brightness settings. Measurements were made with room lights off. Values are the mean of the nine calculated contrasts from values measured across the screen for black and for white squares. MC: Michelson contrast. WC: Weber contrast. All tablets met the requirements of the ICO standard (L_black_/L_white_ ≤ 15%) and of BS 4274 (Weber contrast ≥ 90%).

nominal brightness setting	100%			75%			50%			25%			0%		
	MC	L_black_ / L_white_ %	WC	MC	L_black_ / L_white_ %	WC	MC	L_black_ / L_white_ %	WC	MC	L_black_ / L_white_ %	WC	MC	L_black_ / L_white_ %	WC
iPad 3	98.0	1.00	99.0	99.0	1.00	99.0	97.9	1.04	99.0	98.0	1.02	99.0	98.7	0.65	99.4
iPad 4 #1	98.0	0.98	99.0	99.0	0.98	99.0	97.9	1.00	99.0	98.0	1.04	99.0	98.7	0.60	99.4
iPad 4 #2	98.1	0.94	99.1	99.1	0.94	99.1	98.2	0.92	99.1	98.1	0.97	99.0	99.6	0.20	99.8
iPad 4 #3	98.2	0.92	99.1	99.2	0.92	99.1	98.2	0.90	99.1	98.2	0.92	99.1	100	0.00	100
iPad Air 2 #1	98.8	0.62	99.4	98.7	0.65	99.4	98.7	0.64	99.4	98.7	0.64	99.4	98.6	0.70	99.3
iPad Air 2 #2	98.7	0.67	99.3	98.7	0.66	99.3	98.7	0.64	99.4	98.7	0.63	99.4	98.6	0.70	99.3
iPad Air 2 #3	98.7	0.67	99.3	98.7	0.66	99.3	98.7	0.66	99.3	98.7	0.68	99.3	98.5	0.74	99.3

The mean white luminance of each EDTRS chart varied widely and black letter luminance always exceeded the luminance of tablet black squares, even at maximum tablet brightness setting. Consequently, mean Weber contrast for each chart (96.2–97.1%) was lower than for any tablet/brightness setting combination (Table 3).

**Table 3:** Luminance (cd/m^2^), contrast (%) and luminance uniformity of three EDTRS charts. Measurements were made with room lights on. Values represent the mean of the nine values measured across the chart for black optotypes and for adjacent white areas. All charts met BS 4274 (≥ 120 cd/m^2^) and ICO requirements (≥ 80 cd/m^2^) for luminance and for contrast (BS 4274, Weber contrast ≥ 90%; ICO, L_black_/L_white_ ≤ 15%). Chart parameters which do not meet the BS 4274 luminance uniformity requirements (>80%) are in bold.

	black optotype luminance (cd/m^2^)	adjacent white luminance (cd/m^2^)	contrast L_black_/L_white_	Weber contrast	luminance uniformity L_min_ / L_max_ (%)
chart #1	13	354	3.8	96.2	85
chart #2	9.5	270	3.5	96.5	**70**
chart #3	12	418	2.9	97.1	**75**

#### Compliance with standards

All tablets met the overall luminance requirement of the ICO (≥ 80 cd/m^2^) providing the brightness setting was 50% (half-way) or higher. A nominal brightness setting of 75% or higher was required to meet the BS 4274 requirement (≥ 120 cd/m^2^), although at the 50% setting, mean luminance was only around 0.05 log units below the required level (Table 1). All EDTRS charts met the requirements of both standards (Table 3).

Contrast of the tablets across all brightness settings was ≤ 1.04% in terms of the contrast ratio (L_black_/L_white_) used by the ICO standard, exceeding its requirement to be ≤ 15%. Weber contrast was ≥ 99% for tablets across all brightness settings, exceeding the minimum requirements (≥ 90%) of BS 4274–1:2003. EDTRS charts had slightly poorer contrast, with contrast ratios (L_black_/L_white_) of 3.8%, 3.5% and 2.9% and Weber contrasts of 96.2–97.1%, but still exceeding contrast requirements of both standards.

### Luminance uniformity

Luminance uniformity was generally high for the tablets (room lights off), and was not greatly affected by the brightness setting. The most uniform device (iPad 4 #1) showed less than 10% change in luminance across its surface, whilst the least uniform device, iPad 4 #2, showed around 23% change in luminance across its surface (Table 4, Fig 3). The EDTRS charts (room lights on) all showed greater luminance variation than even the most variable of the tablets: changes in luminance from the brightest to the dimmest part of the chart background measured 15%, 30% and 25% for ETDRS charts 1, 2 and 3 respectively (Table 3, Fig 3).

**Table 4:** Luminance uniformity (white squares, L_min_/L_max_, %) of seven tablets at five different nominal brightness settings. Measurements were made with room lights off. Device/setting combinations which do not meet the BS 4274 requirements (> 80%) are in bold.

nominal brightness setting	100%	75%	50%	25%	0%
iPad 3	84	83	80	83	82
iPad 4 #1	91	91	91	89	90
iPad 4 #2	**77**	**77**	**76**	**77**	**77**
iPad 4 #3	85	86	85	85	82
iPad Air 2 #1	**77**	**78**	**78**	**77**	81
iPad Air 2 #2	80	81	80	80	80
iPad Air 2 #3	85	85	86	85	88

#### Chomparison with standards

Luminance uniformity of the tablets was very close to or exceeded the requirements of BS 4274 (L_min_/L_max_ > 80%). Two of the seven devices did not meet the requirements: one (iPad 4 #2) had uniformity of 76–77% across the five brightness settings, and another (iPad Air 2 #1) had uniformity of 77–81% across the five brightness settings (Table 4).

### Effect of room lighting

Three tablets were re-measured, but this time with the room lights on to emulate a typical visual acuity testing situation. Nominal brightness setting of 75% was used. Switching on the room lights increased the apparent average white luminance of the tablets by about 5% (0.02 log units), but increased the average black luminance by over 200% (over 0.5 log units). Michelson contrast and Weber contrast fell by 3% and by 1–2% respectively, while L_black_/L_white_ contrast ratio increased by 1–2%. Luminance uniformity improved a little for all tablets, bringing all into BS 4274 specification (>80%) (Table 5).

**Table 5:** Pairs of data (room lights off→room lights on) for three tablets. For luminance and contrast, values represent the mean of the nine values measured across the screen for black and for white squares at the same location, with the nominal brightness level set at “75%”. Screen parameters which do not meet the BS 4274 requirements are in bold.

	black square luminance (cd/m^2^)	white square luminance (cd/m^2^)	Michelson contrast	contrast L_black_/L_white_ (%)	Weber contrast (%)	luminance uniformity L_min_/L_max_ (%)
iPad Air 2 #1	1.2→4.3	181→191	98.7→95.5	0.65→2.3	99.4→97.7	**78**→82
iPad Air 2 #2	1.2→4.3	179→190	98.7→95.5	0.66→2.3	99.3→97.7	81→81
iPad Air 2 #3	1.1→3.8	166→174	98.7→95.7	0.66→2.2	99.3→97.8	85→89

All three charts were re-measured with the room lights off. This effectively measures the intrinsic luminance of the devices, decreasing the apparent average white luminance of the EDTRS charts by 32% (0.17 log units), and decreasing the average optotype luminance by 59% (0.38 log units). All charts continued to meet both the ICO (≥ 80 cd/m^2^) and BS 4274 (≥ 120 cd/m^2^) requirements. Weber contrast was slightly higher with the room lights off. With room lights *on*, luminance uniformity improved for two charts, bringing one chart into BS 4274 specification (>80%), and was unchanged for the third chart (Table 6).

**Table 6:** Pairs of data (room lights off→room lights on) for EDTRS charts. For luminance and contrast, values represent the mean of the nine values measured across the chart for black letters and for adjacent white areas. Chart parameters which do not meet the BS 4274 requirements are in bold.

	optotype luminance (cd/m^2^)	white luminance (cd/m^2^)	contrast L_black_/L_white_ (%)	Weber contrast (%)	luminance uniformity L_min_/L_max_ (%)
chart #1	5.2→13	244→354	2.1→3.8	97.9→96.2	**76**→85
chart #2	3.1→9.5	174→270	1.8→3.5	98.2→96.5	**66→70**
chart #3	6.6 →12	301→418	2.2→2.9	97.8→97.1	**75→75**

### Summary

Two standards describe the photometric qualities required of visual acuity test charts [20,21]. We have shown that seven tablets, measured in a dark room, exceeded the standards’ requirements for mean luminance providing the nominal brightness setting is above 50%, and exceeded the standards’ requirements for contrast regardless of brightness setting. Two of the seven tablets fell marginally short of the required luminance uniformity threshold. Re-assessing three tablets at a nominal 75% brightness setting and with room lights on made little difference to mean luminance or to contrast, but all three tablets then exceeded the luminance uniformity threshold, where one had previously failed. We have also shown that three typical, clinical standard ETDRS charts in a well-lit room exceeded the standards’ requirements for mean luminance and for contrast, but two charts fell short of the required luminance uniformity threshold. With room lights off, mean luminance and contrast remained adequate, but all three charts then failed to meet the luminance uniformity requirement. Tablets showed much less inter-device variability, higher contrast, and (under room lighting) better luminance uniformity than charts, providing they were operated at suitably high brightness.

## Discussion

The iPad tablets tested here were more compliant with British and international photometric standards for vision testing than the retro-illuminated ETDRS charts currently used in a tertiary referral dedicated ophthalmic unit. Standards for luminance and for contrast were met by both tablets and charts, but charts generally had lower contrast and greater variability in both luminance and contrast. The mean luminance fluctuations measured here (for example, tablets at 75% brightness varied from 165–202 cd/m^2^, and charts ranged from 270–418 cd/m^2^) are very unlikely to affect clinical measurement: even doubling chart luminance in this range improves acuity by just 1 letter optotype in a line [18,24]. Weber contrast varied between tablets (at 75% brightness, for example) from 99.0–99.4%, and varied between charts from 96.2–97.1%.

Luminance uniformity was 77–91% for the tablets (75% brightness, room lights off), in good agreement with findings elsewhere for an iPad 3 screen of variation from -5 to -23% relative to screen centre [6]. Luminance uniformity was slightly poorer and more variable for the charts (70–85%, room lights on). The adverse effect of uneven luminance would be to create zones where the contrast of the optotype relative to its background differs substantially from other areas of the test surface, introducing greater error in clinical thresholds, or even elevating thresholds. However, for both tablets and charts, the cause of the luminance non-uniformities (uneven-ness of either intrinsic screen brightness of extrinsic lighting) affects both black and white areas, and contrast is thus relatively unaffected (see Fig 3, right hand diagrams), as found elsewhere [6]. Indeed, given the plateau observed in the relationship between luminance and acuity within the range of chart luminance, the stringent criterion referring to uniformity in BS 4274–1:2003 is difficult to explain from a clinical perspective for high contrast acuity. However, this standard may be relevant for contrast sensitivity testing, where the additive effects of luminance and contrast are important, and large discrepancies are reported in the test results between testing modalities, particularly under varying lighting conditions [25].

### Study limitations

We made no effort to control for the effects of battery power. Other studies have shown screen luminance stability of the first generation iPad, maintaining 275 cd/m^2^ from 100% to 5% battery charge [7]. For the iPad 3 on battery power, only a small luminance loss was detected immediately after switching the device on, which was more pronounced if the device was switched off for longer periods [6].

We did not evaluate variability in luminance with viewing angle, instead measuring luminance perpendicular to the tablet or chart surface [23]. Viewing angle is of particular relevance to the tablet platform, given angular effects are likely to be more pronounced when testing is at a closer range. This parameter has been investigated by Parry et al, who tilted photometric devices to mimic the changing viewing angle of the human eye. Testing was performed on the iPad 3 (Apple inc) [6] and also a Google Nexus 10 (Google Inc.) and Galaxy Tab 2 10.1 (Samsung Electronics) [12]. The mean contrast changes over the peripheral areas of the screen varied between devices, but in terms of contrast, the impact was minimal, at most around 1%. The authors asserted that this would be unlikely to be clinically perceptible, as it is less than one just-noticeable difference (JND).

It was not the aim of the current study to undertake any acuity measurements, but it is probable that tablet screen reflections do present an obstacle not present with ETDRS charts. A masked diagnostic study of visual acuity using a first generation iPad showed scores were vulnerable to glare, but including anti-reflective screen covers and positioning to avoid reflections removed the effect [7]. Of note, later generations of tablet screens claim to incorporate anti-reflective coating [26]. Further study is merited to evaluate if such changes in screen technology negate the need for such adaptations.

Aging of tablet screens and of fluorescent bulbs used to illuminate ETDRS charts affect luminance, and possibly luminance variability, and no attempt was made to control for this. An ETDRS chart manufacturer cites lamp life as 9000 hours [27], which equates to an approximate lifetime of 4.3 years of usage assuming use for 8 hours per day during a working week. They further advise annual lamp replacement when cabinets are used for research purposes. In the present study, ETDRS charts #2 and #3 exhibited the poorest luminance uniformity and had been in active clinical use for over 6 years without lamp replacement, falling outside the recommendations made for research purposes. The authors recognise this limitation, and it is likely that the aging of the bulbs has impacted on the results for these charts. However, the most uniform ETDRS chart (#1) was the newest, having been used for 2 years and less than 9000 hours in a clinical trials unit, meeting manufacturers recommendations [27]. Even this chart failed the BS 4274:2003 criterion for luminance uniformity when tested with the room lights off. All charts, however, easily exceeded mean luminance and contrast requirements, again highlighting that the BS 4274:2003 criterion for luminance uniformity may not be adequately evidence-based. All the iPad Air 2 devices were less than 6 months old, with variable usage. The oldest tablet was the iPad 3, which was over 3 years old and in daily use for both recreation and clinical testing: its parameters fell within the range of the other six devices, suggesting that age may not be a major factor in photometric compliance.

Another consideration when applying standards set for charts to tablet devices relates to the significant differences in size of the test area in both platforms. The angular field of view from a tablet device, together with the extent of the background relative to the optotypes, represents an intrinsic difference that could effect clinical results. In a recent study comparing a mobile phone-based single-optotype tumbling E test on the 4.8 inch screen of the Samsung S3 handset (Samsung Electronics), with the ETDRS chart, 272 patients were assessed using both platforms [3]. An average difference of 0.011 logmar was detected (95% limits of agreement -0.31 to 0.42), suggesting that the impact of overall test area is unlikely to be a clinically significant limitation.

### Clinical relevance

The present study suggests that photometric standards of luminance, contrast, and luminance uniformity are met more effectively by the iPads under test (generation 3 to most recent model, the iPad Air 2), than by the dedicated ETDRS charts in active clinical use. The adjustable nature of the nominal brightness setting on such devices allows a relatively simple calibration process. The study fulfilled its aim of documenting the suitability of this mobile technology with reference to high contrast visual acuity test standards, and provides a practical guide to health care professionals working in this field. Specifically, setting the nominal brightness setting to 75% optimised the iPad with respect to ICO and British Standards recommendations (BS 4274–1:2003). These findings are naturally time-limited: due to rapidly changing technology, updated versions reach the market within around two years of the preceding model. Assertions that are valid for the latest platform may not be directly applicable to the next. Furthermore, device manufacturers control the legacy of the incorporated software and hardware without any necessity to conform to standards for chart design, which means it is possible for devices of the same name and generation to have different screen properties. Nevertheless, given that wide fluctuations in luminance result in relatively small changes in target contrast, and consequently small effects on acuity scores, the current data support the view that the increasing use of tablets and similar devices is not likely to be unsafe for clinical high contrast acuity measurements provided that simple checks, based on the adjustable brightness of each device, are conducted.

Healthcare is approaching an impasse where low-cost mobile devices designed for recreation will contain sophisticated assessment capability significantly in advance of those of hospitals and health centres. For vision, clinical standards represent a crucial reference to guide healthcare scientists and clinicians, but the ever-changing digital technology landscape challenges standard setters. Unregulated or inaccurate software/hardware combinations which purport to provide diagnostic information risk harm to patients, yet failing to adopt the best of these technologies risks missing opportunities for novel or lower-cost techniques to detect visual impairment. Robust scientific validation, broadening the remit of international standards, and provision of practical guidance represent possible ways we can maximise the safe adoption of this ubiquitous technology.
